# Moving Beyond “Risky Behavior”: A Qualitative Interview Study Exploring Geosocial Networking App Use by Sexual Minority Men and Women in the UK and USA

**DOI:** 10.1080/19317611.2025.2536248

**Published:** 2025-07-28

**Authors:** Hannah C. E. Madden, Hannah Timpson, Jean M. Breny, Vivian D. Hope, Lorna Porcellato

**Affiliations:** a Faculty of Health, Innovation, Technology and Science, Liverpool John Moores University, Liverpool, UK; b Public Health Department, Southern Connecticut University, New Haven, CT, USA

**Keywords:** Women who have sex with women, men who have sex with men, sex-positive, dating apps, bisexuals

## Abstract

**Objective:**

Research on use of geosocial networking (GSN) applications (e.g. Grindr, Tinder) by sexual minorities has primarily focused on risky behavior and negative health outcomes (e.g. sexually transmitted infections/HIV, drugs, alcohol, violence) among men who have sex with men. Taking a sex-positive approach, this study aimed to understand how sexual minority GSN app users in the UK and USA perceive impacts on their health and how they manage potential risks. Differences between countries and genders are explored.

**Design and methods:**

Qualitative, cross-cultural study in Merseyside (UK) and Connecticut (USA). Photo-elicitation (fake dating profiles) was used in semi-structured interviews conducted with app users seeking same gender partners (n = 31; 15 women, and 16 men) in 2018–2019. Participants were recruited through local LGBTQ+ organizations, social media and from a previous survey, within a quota sampling framework. Transcripts were analyzed using reflexive thematic analysis.

**Results:**

Perceived positive health impacts included social and romantic/sexual connections, boosts to self-esteem, and pleasurable sexual experiences. Some negative outcomes were reported, mainly by men, including rejection and low self-esteem, and racism and discrimination. Participants of all genders used strategies to reduce risks to health. Women were particularly cautious of men on apps. No differences between UK and the USA were noted.

**Conclusions:**

GSN apps can enable positive sexual experiences and have the potential to increase social cohesion and improve mental wellbeing for stigmatized sexual minorities. All participants took measures to protect both their physical safety and mental wellbeing. Using a sex-positive health promotion approach could empower app users to build on their personal strengths and resources. Although GSN app companies may need to do more to tackle discrimination, apps show a promising opportunity for reducing isolation and health inequities. The similarity of the findings in both areas suggests evidence from the USA may be applicable in the UK.

## Introduction

### Lesbian, gay, and bisexual users of GSN apps

Lesbian, gay, bisexual, trans, and queer (LGBTQ+) populations have always been at the forefront of ways to build community, creating independent venues, and utilizing novel technology (e.g. chatlines, the internet, then smartphone apps) to meet other LGBTQ+ people (Ackroyd, 2018; Grov et al., 2014). The first geosocial networking application (GSN app), Grindr, was launched in 2009 and was aimed exclusively at men who have sex with men (MSM). There are now a wide variety of GSN apps (also known as “dating apps”) available for MSM (e.g. Scruff, Jack’d), women who have sex with women (WSW; e.g. HER), and all people regardless of sexual orientation (e.g. Tinder, Bumble). LGB persons and those who report “same-sex sexual partners” are more likely to use GSN apps than their heterosexual peers (Castro et al., 2020; Rogge et al., 2020; neither study report sexual orientation by gender so we cannot compare sexual minority groups).

It is well-established that globally LGBTQ+ people experience worse physical and mental health, often due to discrimination and minority stress (Booker et al., 2017; Meyer, 2003; Zeeman et al., 2019). Since the introduction of Grindr there has been substantial research into how MSM use GSN apps and potential impacts on health. App use is common; between 47 and 76% of MSM in USA have *ever* used an GSN app (Bineau et al., 2021; Gibson et al., 2022; Macapagal et al., 2018). Data from the UK are limited, but suggest levels of GSN app use are likely to be similar to the USA (McGarty et al., 2021). Globally, GSN app use has increased as smartphones have become more common. Thus, even three-quarters may be an underestimate of the proportion of MSM in the UK and USA who have ever used apps. There are no figures on the proportion of WSW who use GSN apps.

There is growing evidence demonstrating associations between GSN app use and negative health and wellbeing outcomes for MSM; including sexual health and HIV risk (Choi et al., 2017; Wang et al., 2018; Zou & Fan, 2017), body image and ill-mental health (Filice et al., 2019; Zervoulis et al., 2020), harassment, racism, and discrimination (Hammack et al., 2022; Lauckner et al., 2019) and drug use and chemsex (Patten et al., 2020; Zou & Fan, 2017). Sexual behavior which is thought of as “risky” (such as condomless anal intercourse, higher numbers of sexual partners, and drug/alcohol use prior to or during sexual intercourse) is higher in MSM who use GSN apps (Choi et al., 2017; Patten et al., 2020; Zou & Fan, 2017).

However, most of these studies do not investigate cause and effect; thus it is still unclear if apps increase risk or if those who take risks are more likely to use apps. Indeed, recent research investigating the link between app use and negative sexual health outcomes for MSM have found *intensity* of sex-seeking behavior^1^ to be a key factor; use of GSN apps does not appear, in itself, to increase risk (Dangerfield et al., 2020; DeVost et al., 2018).

Sexual minority women also experience a plethora of physical and mental health inequalities (Booker et al., 2017; Meyer, 2003; Zeeman et al., 2019), but we know little about how GSN apps may affect their sexual wellbeing or mental health. In contrast to the increasing evidence base about MSM, there has been little research on GSN app use by WSW (Castro & Barrada, 2020). Some evidence shows that WSW are more likely than heterosexual women to have met sexual or romantic partners on the internet or through GSN apps (Cabecinha et al., 2017; Watchirs Smith et al., 2018). The limited research focuses on how WSW manage identity on GSN apps, experiences of discrimination, and how women “stay safe” on apps (Albury & Byron, 2016; Ferris & Duguay, 2020; Pond & Farvid, 2017). There are gaps in the evidence on how women navigate app use, the impacts on their health, or if their experiences are similar to MSM. Investigating app use by WSW, as well as MSM, is important to enable us to understand their potential impacts on the health of marginalized lesbian, gay, and bisexual communities.

This research study focuses on people who identify as lesbian, gay, bisexual, pansexual, queer, asexual, or who use apps to find “same-gender partners”. Although trans and gender diverse participants are included, this research focuses on sexual orientation; transgender identity was not salient to this study. All participants identified as either men or women and used their own gender identity terms when determining what “same-gender” attraction meant to them.

Drawing on the Döring et al. (2021) typology of sexual interactions in digital contexts, this research mainly focuses on sexual interactions *through* digital technologies (interpersonal sexual interactions in face-to-face contexts that were initiated on GSN apps), and to a lesser extent sexual interactions *via* digital technologies (interactions such as cybersex and sexting with strangers conducted through GSN apps).

### Sex-positive approach

The early evidence on the link between ill-health, risk-taking, and GSN app use by MSM has led to a focus on negative health and wellbeing outcomes. The discipline of public health, and to a lesser extent sexual health, tends to focus on preventing negative health outcomes and has been largely dominated by a discourse of risk and danger (Anderson, 2013; Ford et al., 2019; Mitchell et al., 2021). This is particularly true in research with LGBTQ+ groups which mainly focuses on negative elements such as HIV, STIs, and sexual assault (Epstein & Mamo, 2017). Moreover, despite the word “pleasure” being included in the World Health Organization definition of sexual health (WHO, 2006) and the recent World Association of Sexual Health’s Declaration on Sexual Pleasure (Ford et al., 2021), the role of pleasure and positive sexual experiences are often overlooked in health research.

Recently there have been calls for a more sex-positive approach to public and sexual health research, shifting the focus away from danger and disease, toward encouraging pleasurable safe sexual experiences, and sexual wellbeing (Ford et al., 2019; Gruskin & Kismödi, 2020; Mitchell et al., 2021; Pitts & Greene, 2020; Porta, 2020). Sex-positivity acknowledges risks and concerns, yet also emphasizes the importance of sexual pleasure, freedom, and diversity (Williams et al., 2015). A key part of sex-positivity, explained in the framework proposed by Williams et al. (2015), focuses on strengths, assets, wellbeing, and happiness. Using a strengths-based and positive psychology approach like this can empower people to utilize existing resources and capabilities.

In the context of GSN technology it is particularly important for public health research to look at the assets and protective factors for LGBTQ+ people (European Centre for Disease Prevention & Control, 2015; Grov et al., 2014; Williams et al., 2015), including sexual activity. Sexual pleasure and wellbeing are key drivers of sexual activity and cannot be overlooked in health policy, program implementation, and sexual and reproductive health services delivery (Allen & Carmody, 2012; Ford et al., 2019). Sexual satisfaction, sexual self-esteem, and sexual pleasure are positively associated with sexual health, physical health, mental health, and overall wellbeing (Anderson, 2013; Ford et al., 2021). Previous research into how GSN apps affect health and wellbeing has focused mainly on risk and deficit, and we aim to redress this balance.

The research on the benefits of GSN apps in the UK and USA tends to focus on uses and gratifications for use, benefits of the technology over other forms of relationship initiation, identity formation, and GSN app use during COVID-19 lockdowns (e.g. Jaspal, 2017; Miller, 2015; Powell & Powell, 2022; Sharabi et al., 2023).

Some limited research has highlighted positive health and wellbeing outcomes of GSN app use for LGBTQ+ users; particularly in the USA and Australia. For example, mixed methods research in Australia has shown GSN apps are an important space for young LGBTQ+ users to map queer community and find friends (Albury et al., 2019; Byron et al., 2021; Pym et al., 2021) and in the USA GSN apps enable gay and bisexual men to find safe spaces to connect, addressing the lack of gay-specific physical spaces, reducing sexual minority stigma (White Hughto et al., 2017). A recent survey in the USA asked LGBTQIA+ participants “What benefits (if any) have you gained from using dating apps?” (Sharabi et al., 2023). The most common benefits were “Making Offline Connections” (42%), “Expanding the Dating Pool” (21%), “Knowledge and Self-Discovery” (13%), and “Ease of Use” (13%). The responses were similar for men and women. Our study explores these categories further and focuses less on benefits of the technology, and more on how users perceive positive health and wellbeing impacts.

Another mixed methods study in Australia (Hobbs et al., 2017), showed GSN apps enhanced opportunities for connection, had a positive impact on self-esteem and provide a safe space for individuals to explore their sexuality. Hobbs et al. (2017) suggest accounts of dating apps are too pessimistic, and downplay the positives. However, it is important to note this Hobbs et al. (2017) study included heterosexual and LGBTQ+ participants and their findings are not broken down by sexual orientation or gender identity.

There is limited public health research on GSN app use by sexual minorities that takes an explicitly sex-positive approach. One recent survey study in the USA examined “sexting” using a sex-positive framework (Graham Holmes et al., 2021). They found LGBQ participants were more likely to have sent an explicit sext and to report positive self-assessments after sending sexts. However, this study focused on any act of sexting (often between long-term romantic partners) not just GSN apps.

To improve health promotion we need a deeper and more nuanced understanding of GSN apps, enabling us to avoid judgmental health promotion that focuses solely on risks and deficits. The narrative in public health that GSN apps are risky, as well as the negative media coverage (McCosker et al., 2019), may be increasing stress for this already stigmatized group; both by causing users worry and by creating the view within society that only irresponsible risk-takers use these apps. Nonjudgmental health promotion that includes discussion of pleasure and positive elements of sex, has been shown to be effective (Scott-Sheldon & Johnson, 2006; Zaneva et al., 2022).

The majority of public health research on GSN app use by sexual minorities has tended to focus on risk-taking behavior, measured negative sexual disease outcomes and identified potential dangers to mental health. Therefore, we take a sex-positive approach to GSN app use by sexual minorities, exploring a variety of perceived health and wellbeing impacts, as well as the resources and skills people use to increase their sexual wellbeing and maintain personal safety. This approach can help policy makers and health professionals understand people’s existing capabilities, strengths and resources and how they could use these to improve their wellbeing.

### Research context

Cross-cultural comparative health research aims to understand and explain phenomena by describing similarities and differences across areas and can inform the actions of local practitioners and health policy-makers (Øvretveit, 1998). There have been studies with MSM in many countries (predominantly USA, China, Australia, and recently in mainland Europe), however we were unable to find any research that compared GSN app use by sexual minorities in multiple countries. Studies from the UK are limited and, considering the legal and socio-cultural variation between countries, it is not clear if the international evidence is relevant in the UK. Comparing experiences in the two countries allows us to provide a more comprehensive understanding of behavior on GSN apps and identify any differences between cultures. Understanding these variations can help us tailor health promotion interventions to be more culturally appropriate and effective (Al-Bannay et al., 2014). Legal structures and protections, and the development of these, varies greatly between countries, and there are also marked differences in queer culture between countries. Therefore, the use of GSN apps, and context on which these are used, may vary between countries. Evidence may therefore not be generalizable between countries.

The context for this study, Merseyside (MS; a county in north-west England) and Connecticut (CT; a state in north-east USA) have relatively similar LGB rights, legislation and politics, with a combination of affluent and deprived rural areas, small towns, cities with large student populations, relatively small gay scenes, a few LGBTQ+ organizations and annual gay pride events. There are however difference between the areas; the Merseyside population is 91.7% white, whereas 78.8% of Connecticut is white alone (ONS, 2023; US Census Bureau, 2022). The design of health services also differs; a tax-funded free at point of care National Health Service in the UK, compared to the private US health-care system with high point-of-service fees for many users.

Therefore, this qualitative study aimed to use a sex-positive approach to understand (1) the perceived impact of GSN apps on the health and wellbeing of sexual minority users in UK and USA; (2) how these users manage their app use to increase positive outcomes and reduce potential risk and (3) investigate any differences and similarities between the UK and USA and gender groups. This small-scale study is unlikely to elucidate all country-level differences, however, the comparison of two areas will add to our understanding of GSN app use by this population and highlight any major differences among our participants.

## Methods and materials

This was one phase of a wider mixed methods research project on GSN app use (Madden, 2021). Here we report on the qualitative element which was focused on the research questions above. A generic qualitative research approach (Percy et al., 2015) was used to understand experiences and behavior of GSN app users. The study is reported using the consolidated criteria for reporting qualitative research (COREQ; Tong et al., 2007).

### Recruitment

This study used multiple recruitment methods within a purposive sampling framework. (1) Recruitment adverts were shared on social/broadcast media by a variety of LGBTQ+ organizations in Merseyside and Connecticut; (2) Snowballing – participants shared the recruitment advert in their networks; (3) Some participants who took part in an earlier phase of the research (Madden, 2021), provided their email address and consented to be contacted about this qualitative phase. Potential participants emailed the lead author and were sent the participant information sheet. Three participants were acquaintances of the lead author, but the rest had no prior relationship with her.

The quota sample aimed to recruit 7–8 sexual minority women and 7–8 sexual minority men in each location, with a total of 28–32 participants who use GSN apps. This sample size was informed by previous research on this topic (e.g. Ferris & Duguay, 2020; Filice et al., 2019; Lauckner et al., 2019; Pond & Farvid, 2017) and practical constraints. This sample size would allow us to reach thematic saturation within each quota category.

### Participants

Inclusion criteria were: aged 18 years or over, has used a GSN app in the last 18 months to find a same-gender relationship or sexual partner, resident in either Merseyside or Connecticut and able to give informed consent. Response rate is unknown; however, three participants contacted the researcher but dropped out before the interviews took place (two stopped answering emails and one said they were now too busy). Throughout the results section, we use the identity and gender terms given by the participants themselves. For example, some participants identified as bisexual and said they used apps to meet men and women, whereas others identified as pansexual and said they used apps to meet people of any genders.

### Procedure

The semi-structured, one-to-one interviews were conducted by the lead author (lesbian woman) between Autumn 2018 and Summer 2019 on university premises, in coffee shops and in quiet bars during the daytime. Interview venues were chosen by the participants, and approximately half chose coffee shops rather than attending a university building. For interviews that took place in public these were either in venues suggested by the participant as they felt comfortable, or when the interviewer suggested a venue, they were coffee shops she knew well and that provided quiet semi-private areas for discussion.

These face-to-face interviews were audio recorded. Reflective field notes were taken after the interviews. The interview guide was informed by a literature review and included questions on their experiences on dating apps, how they approached app use and their thoughts on safer sex (see Online Supplementary Files for the interview guide). Interviews lasted between 55 and 110 minutes.

The final section of the interviews used photo-elicitation methods. Photo-elicitation, also called photo interviewing, integrates photos and images into interviews and uses them as a stimulus to examine perceptions, understand behaviors or evoke memories (Schwandt, 2007). Fake GSN app profiles were shown to the participants to prompt further discussion. The inclusion of these images during interviews served three purposes. Firstly, images can prompt memories and encourage more detailed discussion than simple questions (Glaw et al., 2017). Secondly, using the fake profiles enabled the participants to discuss what they would do in particular situations and introduced a useful element of consistency between the interviews (Ritchie et al., 2013). Thirdly, the profiles allowed a more relaxed way to discuss some of the more sensitive topics such as barebacking and HIV; looking at the pictures gave the interviewee a slight break and allowed the participant to feel less “grilled” by the researcher (Banks, 2007).

The fake GSN app profiles were developed from a literature search and review of actual GSN app profiles. Four Tinder profiles were presented to women and four Grindr profiles to MSM. Tinder and Grindr apps were chosen as they were the most popular apps for MSM and WSW, respectively, in an earlier phase of this research. No usernames, demographic information or direct text was taken from the real profiles and Stock Images were used for profile pictures. The fake profiles can be found in Online Supplementary Files.

The interview tools were piloted with one man and one woman (both in MS), and some minor changes were made after discussion with the pilot participants. During the pilot interview with the man, two fake profiles were presented together but this made the conversation confusing as participants pointed, which the interviewer had to explain for the audio recorder. In the second pilot and all subsequent interviews, fake profiles were presented one after another – starting with the “mild” and less controversial profiles proceeding to the more highly sexualized, potentially “risky” profiles later. After the pilot stage the photo on the first women’s fake profile was changed to make her a more neutral, conventionally attractive women. These pilot interviews were included in the analysis as changes were so minor as not to affect results.

### Analysis

Interviews were transcribed verbatim and imported into NVivo 12 Pro (released 2018, QSR International). Data were analyzed using reflexive thematic analysis which involves six steps; familiarization, coding, generating initial themes, reviewing themes, defining and naming themes and writing up (Braun & Clarke, 2006, 2019). Thematic analysis is flexible and inductive, and thus compatible with a generic qualitative approach (Percy et al., 2015).

All codes and initial themes were generated by the lead researcher and involved reflecting on their assumptions and how this may have affected the analysis (Braun & Clarke, 2021). Much public health research has adopted the approach of using multiple coders and assessing inter-coder reliability in qualitative research. However, in this study all the data was coded by only one author. This decision was taken for two reasons. Firstly, the lead author conducted all the interviews and had detailed understanding of the data. The need for agreement across coders can come at the expense of interpretative insight (Keene, 2020). As Morse (1997, p. 446) argues, a second coder may not have the same knowledge base, has not listened to all the interviews and “does not therefore have the same potential for insight or depth of knowledge required to code meaningfully”. Secondly, employing a second coder is resource intensive (Keene, 2020). This study was undertaken as part of PhD research program, and we had to take a pragmatic approach as the use of multiple coders would have been difficult to adopt within the parameters of a cross-cultural study.

With all qualitative research, the researcher has the potential to influence the findings during analysis. The details of the thematic analysis were discussed with coauthors and amended after reflexive discussion at three stages: (1) initial codes were reviewed halfway through coding the transcripts, (2) after initial coding we deliberated how codes could be grouped into themes, (3) during write up, themes were reorganized and discussed again. This process increased confirmability and maintained a distinction between the researcher’s personal values and the participants (Tolley et al., 2016).

The case classification facility of NVivo was used to label all interviews for area of residence and gender; which allowed all codes and themes to be compared. Illustrative quotes are labeled with a pseudonym, participant gender and area. We have used some semi-quantification (i.e. “many”, “a few”, “a minority”) in the results as this helps the reader to understand the patterns, regularities, peculiarities and idiosyncrasies in the large dataset (Neale et al., 2014). It is not meant to convey generalizability beyond the study population, but this semi-quantification can “complement the participants’ perspectives in providing a clearer and more in-depth understanding of what’s going on for individuals who belong to a particular category” (Maxwell, 2010, p. 470).

### Ethical considerations

Ethical approval was given by Liverpool John Moores University, Research Ethics Committee in July 2018 (ref 18/PHI/030). Ethical approval was also granted by the Southern Connecticut State University, Internal Review Board in October 2018 (ref 18–146). All participants provided informed written consent, were free to withdraw and were signposted to support services. To ensure anonymity participants were asked to choose a pseudonym and any identifying names or places were removed during transcription.

## Results

Thirty-one interviews were conducted, 16 in Connecticut, USA (CT; 8 women, 8 men) and 15 in the Merseyside, UK (MS; 7 women, 8 men). A total of 16 men and 15 women (n = 31, median 27 years, range = 19–54 years) took part in interviews. Participants in the two areas were similar ages (CT median = 27.5 years, MS median = 27 years). However, the men participating were younger (CT median = 26 years, MS median = 23.5 years) than the women (CT median = 30.5 years, MS median = 29 years). The majority of participants were white; three of the MS and five of the CT participants identified as another ethnicity (Table 1). In CT, a third (5/16) of participants were students and a third (6/15) worked for a university. In Merseyside more than half of the participants (9/15) were students. People had been using GSN apps for between 2 months and 9 years. Approximately a third of participants in both areas were old enough to have been dating before the invention of GSN apps.

**Table 1.** Participant information (all terms those used by the participants themselves).

Country	Pseudonym	Gender	Age	Relationship status	Sexual orientation	Using/used apps to meet	Approx. time using apps
UK	Aurelio	Man	30	Single	Gay	Men	10 months
	Belinda	Woman	33	Single	Queer	Women	4 years
	Bernard	Man	30	Single	Gay	Men	9 years
	Bridget	Woman	33	Single	Bisexual	Men and women	2 years
	Courtney	Woman	37	Monogamous relationship	Gay	Women	1 year
	Fitz	Woman	27	Single	Lesbian/queer	Women	3 years
	Lollipop	Man	20	Single	Bisexual/pansexual	Men and women	3 years
	Luffy	Man	21	Single	Gay	Men	1–2 years
	Mickey	Woman	29	Single	Lesbian/gay	Women	2 years
	Natalie	Woman	24	Non-monogamous relationship	Lesbian	Women	6 years
	Phil	Man	20	Single	Gay	Men	2–3 years
	Sam	Man	20	Single	Homosexual	Men	2 years
	Savana	Woman	19	Single	Bisexual	Meeting women, talking to men	6 months
	Steve	Man	26	Single	Gay	Men	5 years
	Yellow	Man	38	Non-monogamous relationship	Gay	Men	9 years
USA	Akina	Woman	24	Single	Pansexual	Any gender	1–2 years
	Alex	Woman	51	Dating someone	Lesbian	Women	3–4 years
	Barbara	Woman	54	Monogamous relationship	Gay or lesbian	Women	9 months
	Candide	Woman	25	Monogamous relationship	Bisexual	Men and women	8 months
	Dee	Woman	25	Non-monogamous relationship	Bisexual	Men and women	4 years
	Emma	Woman	30	Single	gay/lesbian	Women	6 years
	George	Man	26	Single	Queer/gay	Men	8 year
	Jammal	Man	22	Single	Gay	Men	5 year
	Jesse	Man	31	Single	Gay	Men	9 years
	John	Man	20	Monogamous	Gay	Men	2 years
	Patrick	Man	22	Single	Gay	Men	3 years
	Peter	Man	29	Dating a few people	Homosexual	Men	2–3 years
	Sebastian	Man	31	Dating someone	Gay	Men	7–8 years
	Soot	Woman	31	Single	A-/demi-/bi-sexual/queer	Any gender	2–3 months
	Topher	Man	26	Single	Gay	Men	6 years
	Zoe	Woman	42	Single	Asexual	Women	6 months

**Figure 1.**
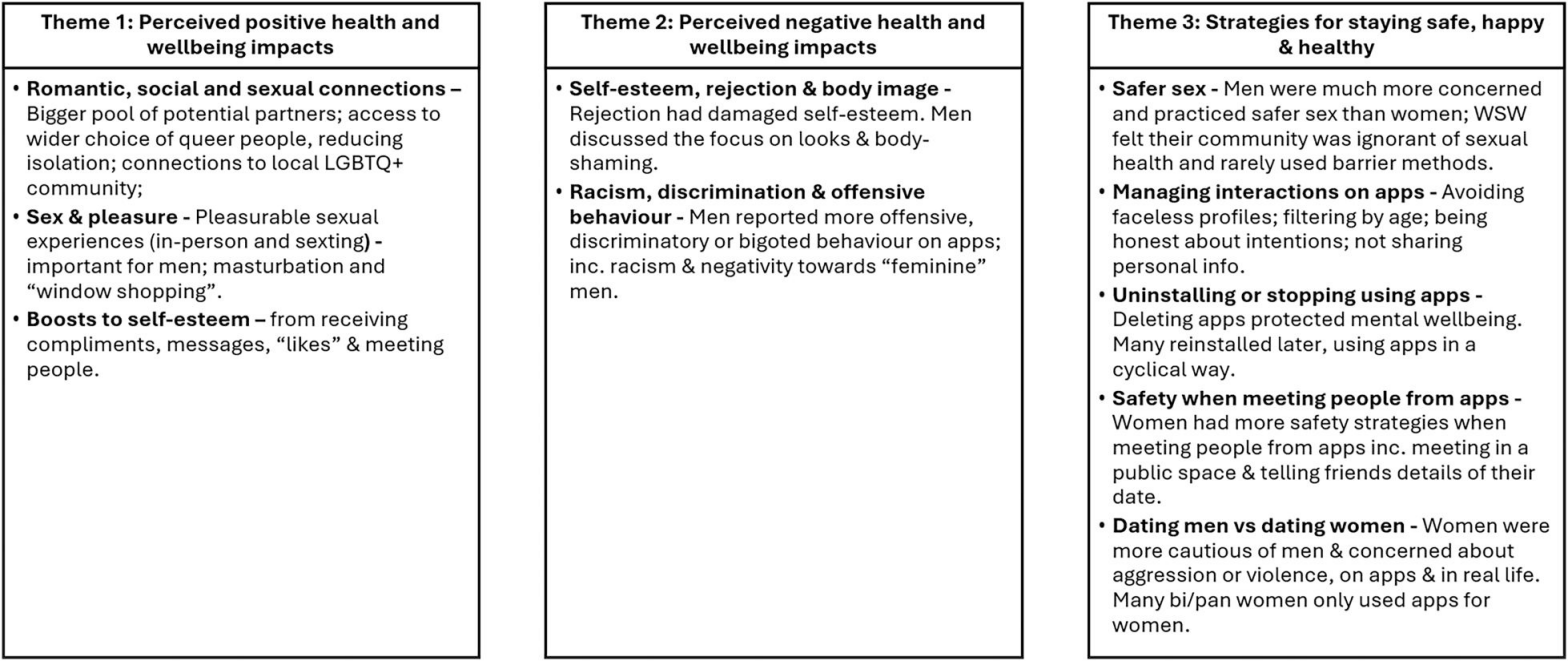
Themes and sub-themes.

Three main themes were generated: (1) perceived positive health and wellbeing impacts; (2) perceived negative health and wellbeing impacts and (3) strategies for staying safe, happy, and healthy. Within these, several sub-themes were generated; displayed in Figure 1.

### Theme 1: Perceived positive health and wellbeing impacts

All participants reported positive experiences and benefits of using GSN apps. Within this, three sub-themes were identified: romantic, sexual and social connection, sex and pleasure and boosts to self-esteem. The only notable gender differences were in the sex and pleasure sub-theme which was considered more important for men than women. For all other sub-themes, only minor nuanced gender differences emerged. For example, all genders talked about using apps when bored; however, men tended to associate feeling horny with being bored, whereas women never linked the two emotions. No differences between the two areas were identified. Generally, participants reported more positives of app use than negatives.

### Romantic, social, and sexual connections

One of the main perceived benefits of GSN apps was that they increased the pool of potential partners, and helped users find queer people they were unlikely to meet otherwise. Many participants of all genders discussed the GPS function of apps and the location of potential partners, with those who were looking for something casual wanting to find people in their city or close by.

*…gay women, like everyone knows their fucking ex’s fucking best mate, whatever, you know, it’s not uncommon to date the same woman as somebody that you know. Whereas in the straight community it would be fucking unheard of…Yeah so it enlarges the pool. (Belinda, queer woman, MS)*

Love and long-term relationships were an important positive outcome of apps. Many participants discussed how they’d had a serious relationship with someone they had met on an app; some had been seeking this whilst others were not actively looking for a long-term relationship when they met someone and it turned serious.

*actually it was just a [Grindr] hook-up and then we got on quite well…we both felt embarrassed because it’s obviously like a sex app…So we were like “it’s a bit weird but do you want to go for a drink, like and maybe just not have sex?” [laughter] (Sam, homosexual man, MS)*

In both areas, participants of both genders reported their app use had also led to satisfying and enjoyable non-sexual, social relationships. Many participants talked about feeling lonely or isolated at some point and using apps to try to reduce this. For those who had just “come out” or were exploring their sexuality, apps were an important way to meet local LGBTQ+ people and helped them feel a sense of a community. Some participants reported making friends, increasing social networks or finding a queer community through apps; many reported friendships were a bi-product of app use discussing how they stayed friends with someone they had dated or hooked-up with.

*I guess you get to meet people. And if it isn’t going to develop into anything else you get to keep them as a friend if they are funny or feel like they could be a good friend… (Mickey, lesbian woman, MS)*

### Sex and pleasure

A common positive outcome of apps was that they are good to kill time when bored, or to entertain themselves with no intention of meeting anyone; referred to as “window shopping”. There was a nuanced difference between men and women; men were more likely to talk about a state where they were both bored and sexually aroused (“horny”) at the same time; no woman talked about these two states co-existing.

*You wake up at 4am and you’re bored, you’re a little horny. And you can just you can talk to anybody across the world…It’s kind of fun. You can go to profile have a good time… (Jammal, gay man, CT)*

Many of the men interviewed discussed how sexual activity and pleasure was a positive outcome of using GSN apps. There was a marked difference between the genders, as no women discussed these as a specific benefit of apps.

*Interviewer: The other positives, what do you get out of [app use]?*

*Lollipop: A lot of ass.*

*Interviewer: [laughter] Sex?*

*Lollipop: Sorry yeah [laughter]. (Lollipop, bi/pan man, MS)*

Using apps for “sexting” (exchanging explicitly sexual messages or pictures) with other app users they were unlikely to meet was often discussed by men. Sexting was seen as an enjoyable part of app use for many men. No women discussed using apps for exchanging images with app users they had not yet met.

*I’ll still like sometimes log on to chat with people. Usually if I’m like going to masturbate …I like do that with people. And usually I use Scruff for that because you can talk to people who aren’t local to you. (George, gay man, CT)*

### Boosts to self-esteem

It was very common for participants of all genders to discuss how using GSN apps could boost their self-esteem and wellbeing when they received compliments or got “likes” or messages from attractive app users. For most, apps made them feel like others desired them, which made them feel good about themselves. However, apps could also damage self-esteem; half of participants discussed apps having both a positive and negative impact on their self-esteem and for some it was a challenge to balance this (see theme 2)

*…it’s really nice for your ego. It’s a nice boost…instant gratification, flattery, almost instant. (Candide, bi woman, CT))*

### Theme 2: Perceived negative health and wellbeing impacts

As well as the positive impacts of app use, participants had also experienced a variety of negative effects on their wellbeing. Many participants experienced these negative outcomes alongside the reverse positive outcomes. For example, apps were thought to have both a beneficial and a detrimental impact on self-esteem. There were some differences between men and women on these sub-themes; mainly men reported more experiences of body shaming, racism and prejudice on apps.

### Self-esteem, rejection, and body image

As mentioned above, half of participants discussed how apps had both a positive and negative impact on self-esteem. However, many participants of both genders felt GSN apps had damaged their self-esteem or dented their ego at some point. Ghosting (when someone suddenly ends a relationship and without explanation, not replying to any communication and often blocking them on all platforms; Freedman et al., 2019) was particularly hurtful and insulting. However, participants also found it distressing to have their messages ignored.

*What I didn’t like [about apps] is you have to have a very thick skin…., so, I would say “hi, I read your profile, we seem to have this in common, would you like to talk?” Nothing, nothing, nothing” (Barbara, gay/lesbian woman, CT)*

Men talked about body image, how apps made them feel about their looks or discussed body-shaming on apps. Most of the men thought apps were too image focused; this reflected the gay scene in real life but was amplified by the design of apps, which places photographs at the forefront and prioritizes visuals over text. Most men had seen judgmental statements on apps profiles (e.g. “no fat people” or “fit guys only”) and found these offensive, though not as bad as racist statements (see below). Comparing their bodies to other men on apps also had a negative impact on self-esteem.

*I think it’s not always a very kind space…I think the anonymity lets people feel a little bolder in being rude…people are more callous with their words. (George, gay man, USA)*

*How could you not take it personally on some level when somebody literally just rejects you because they don’t like the part of your body…I think being on Grindr is like going to a butchers for some people. “Oh that is a nice slab of meat, oh that one has too much fat on it” (Phil, gay man, MS)*

However, among women body image was discussed much less often; either in terms of how their own self-esteem issues were exacerbated by apps, or they were concerned about how their physical attractiveness would be viewed by other app users. No women had received any negative comments from women on apps, but some plurisexual women had seen statements on men’s profiles about preferred body size and on women’s profiles about style or body type.

*Men looking for women, sure, they never want fat women. There’s so much fat phobia, like tonnes of fat phobia. (Dee, bi woman, CT)*

### Racism, discrimination, and offensive behavior

Generally, men had more experiences of offensive, discriminatory, or bigoted behavior on apps; many of which negatively affected their wellbeing even before they met up with someone. Almost all men in both CT and MS had seen racism on Grindr. Examples ranged from statements such as “prefer white guys”, to profiles explicitly stating “no Asians” or “no blacks”. No women had seen any statements about ethnicity on women’s GSN app profiles.

*I always feel like there’s a lot of hatred on the apps, there’s a lot of, you know, like the racism and that to me is just so disgusting, I hate seeing that… I will look at the profile and if I see any of that I will block them immediately. (Sebastian, gay man, CT)*

Amongst the men, many discussed seeing many statements such as “straight acting”, “masculine men”, “no femmes” or “no camp”. These sorts of statements were viewed as discriminatory, homophobic, and made participants quite angry. Some participants interpreted these statements as a form of internalized homophobia from men who were reluctant to identify as gay or bisexual.

*“hot straight guys” for one, that pisses me off straight away. It’s just this whole like gays being homophobic sort of thing. As in this kind of, like, “camp isn’t desirable I want a straight guy”. He’s not fucking straight if he’s fucking your ass”. (Steve, gay man MS)*

Some men (and women who dated men) felt the anonymity of the online space allowed users to be more direct, bad mannered and rude with their requests. The lack of face-to-face contact allowed people to disregard normal social rules and behave in a way they would not in real life.

*I’d say because Tinder, Grindr, Bumble, literally any of those kind of ones all kind of, how do I put it, they come off so, not aggressive but it’s all too body shaming…. (Jesse, gay man, CT)*

### Theme 3: Strategies for staying safe, happy, and healthy

Participants of all genders used strategies to reduce risk and protect their health and wellbeing both when using apps and when meeting people from apps. Because of these methods to reduce risk and increase good experiences, generally participants reported the positives of app use outweighed the negatives. Within this theme there are five sub-themes: safer sex; managing interactions on apps; safety when meeting people from apps; uninstalling or stopping using apps, and; dating men compared to dating women. There were no differences between areas, however there were some marked gender differences especially in relation to safer sex and personal safety.

### Safer sex

There were notable differences between how genders approached safer sex, though no differences between areas. Although men were concerned about safer sex and talked about how they felt condom use was important, during the interviews most men did discuss that they did not use condoms consistently with all partners. Some men discussed how they did not always use condoms with casual partners; however most of these made informed decisions about risk (e.g. they were on PrEP, did not have anal sex, or asked their partner about when they were last tested). A minority of men reported they had condomless anal sex with regular partners they trusted.

*Topher: if I’m getting intimate with somebody would we talk about putting on a condom?…yeah condom, always condom.*

*Interviewer: is it done or is it talked about?*

*Topher: it’s just done…I mean a couple of times it hasn’t happened but…[laugher] (Topher, gay man, CT)*

Most men felt confident to talk about condom use with casual partners; sometimes these conversations happened on the app before meeting up but usually they were had in person just before sex. Those on PrEP were the only participants who seemed to be more open to condomless anal sex and did not assume condom use was the default. The topic of “barebacking” or “raw” sex (a deliberate decision not to use a condom to improve physical pleasure during anal sex) was discussed by most men. More than half of these men said they would actively avoid app profiles that mentioned “BB”, or “raw” sex and most interpreted such statements as warning an app user was risky and may expose them to HIV or STIs.

*This [pointing at BB abbreviation on fake profile] is something I don’t…and even mentioning that’s just “no, turn off”. (Peter, gay man, CT)*

Women were generally less concerned about safer sex. Many women discussed how WSW usually assume sex between two women has no risks. Most acknowledged their dismissal of risk and accepted that, yes, women could transmit STIs, but that generally they forgot about this or did not consider it when having sex or meeting female partners. A third of women had never had a conversation with a female partner about safer sex and some participants questioned how risky unprotected sex between women was, as they did not know any information about STI prevalence or the physical risk of transmission.

*Probably really bad because we can get STIs [laughter]…but I have never worried…Never used protection…I’ve never had a conversation with any sexual partners, no. (Mickey, lesbian women, MS)*

Only a third of women reported having used protective measures with women including using condoms on sex toy, and washing or not sharing sex toys. Barrier protection during skin-to-skin contact was very rare; only two women brought up using dental damns for oral sex and one woman reported ever using latex gloves.

*…the thing that people are careful about is like sharing toys. But yeah, dental dams. I feel like, it’s…show me data proving that it makes me any, like…any significant percentage safer and I would consider using it…I don’t know…like I feel like sex is more like creative or something with women. (Emma, gay/lesbian woman, CT)*

Of the seven plurisexual women, most talked about how they were more concerned about sexual health and practiced safer sex when with men, compared to women. These women would insist on condoms with men, and some were on the contraceptive pill. Safer sex was the norm with male partners; however, most were less concerned about sex with women and rarely used any barrier methods or discussed sexual health screening.

*Dee: I always brought [safer sex] up. After [I was diagnosed with an STI] I always brought it up. I always asked if they were tested. It didn’t always stop me from proceeding but I always asked.*

*Interviewer: And women as well?*

*Dee: Yeah, er, no. I never asked women actually (Dee, bi woman, CT)*

### Managing interactions on apps

All participants discussed “faceless profiles” (GSN app users who do not include a picture of their face online), although they were more common on men’s apps. Most participants actively avoided faceless profiles as they could be a “catfish”; all participants said they would not meet up with someone without ever having seen a photo, although a minority had in the past. Some men suspected app users who did not have face photographs were in the closet, “on the down low” or secretive about using GSN apps.

*when you don’t show your face you’re hiding something…at least when you’re willing to show your face, whatever your intentions are, at least you’re showing it and hopefully you’re not using a fake profile…Either they’re discreet, as in they don’t want people to know that they’re on the website or they’re not who they say they are. (Sebastian, gay man, CT)*

The majority of participants had rules about what aged people they would date; two thirds stated specific minimum and maximum ages they would consider dating, and therefore who they are likely to talk to on GSN apps. The majority of participants brought up authenticity and being truthful about intentions; they wanted others to be honest and they were upfront about what they were looking for on apps. This honesty enabled them to be considerate about other people’s feelings and ensure there was no confusion.

*it’s more common to meet someone who is not exactly on the same page as you…if I’m not going to invest emotionally, and this person seems emotionally investing in me, then it is my responsibility to let them know where I am at in the nicest possible way. And also perceptive of their needs (Soot, pan woman, CT)*

Another common safety strategy when using apps was to not to share too much personal information, for example not giving people their phone number or link to social media too quickly. This was a way to protect themselves and avoid potential harassment.

### Uninstalling or stopping using apps

Another common coping strategy and a way to protect mental health was taking the decision to stop using or uninstalling GSN apps. Many participants discussed how they had decided to delete an app after becoming annoyed with it, receiving negative and creepy comments, or becoming frustrated about how it made them feel. Some people discussed deleting apps as a reaction to specific experiences of harassment, usually from men, and some people said they took “mental health breaks” from apps. Many people used apps in a cyclical way, downloading them and using for a while, getting frustrated or unhappy on apps, then having a break, just to come back to the apps a few weeks or months later and find they had a similar experience.

*You get, like, disappointed or disrespected enough times to be like “fuck this, I’m deleting it” and just for enough time to forget and then you re-download it and then as soon as you go back you’re like “oh my fucking god, why did I do this?” (Akina, pan woman, USA)*

One participant (a gay man in CT) reported he had stopped using apps because he didn’t like them; all other participants who no longer used apps had ceased use because they started a monogamous relationship. Generally participants reported the positives of app use, outweighed the negatives.

### Safety when meeting people from apps

Generally, women discussed more safety strategies when meeting up with people from GSN apps. The two main strategies were meeting in a public place and telling friends about their date.

*I told my sisters where I was going, I sent them the address so they knew…No houses. And so like hotels, bars, restaurants. Oh, like just like a place that was public, open. I had a set amount of time that would spend with them. So I’d always message my sister “This is where I’m going today, this is the person’s name and if I don’t message you by 4 o’clock then ring me” (Courtney, gay woman, MS)*

Some participants (mainly men and plurisexual women) talked about “murderers” or being killed or attacked on a date; the men usually talked in a jokey tone of voice but with genuine concern. A small number of people (men and women) discussed experiences of non-volitional sex with people they met on apps – although none attributed it to the app itself. One man also reported being robbed by a man he met on Grindr.

*Pretty much every time I go to meet someone [from an app] or someone’s coming around to meet me I’m always thinking “is this when I’m gonna die?” [laughter] (Luffy, gay man, MS)*

### Dating men versus dating women

Most women talked about the differences between dating men and dating women; both in terms of how they interact in the app and how they approach meeting up in real life. All of the plurisexual women felt men were generally more forward, “creepy” and threatening on all apps. Therefore, all the women who also dated men were much more guarded; some specifically set apps to exclude cis men, and some said they would never meet up with a man from apps.

*I don’t think I’d meet a man through [apps]…Erm, again, me generalising but, like, predatory behaviour, just all those straight dudes seem to be twats… (Savana, bi woman, MS)*

All the women who used apps to meet men were much more cautious when meeting up with men in real life, compared to when they were meeting women, as they were more concerned about violence, threats and aggression. Some women talked in disbelief about what it must be like to feel safe to go to the house of a man they just met on an app, just to hookup.

*that’s the difference that comes with men and women…I feel much more comfortable meeting women off of dating apps and therefore I’ve met more women off of dating apps…And with men [I don’t know] you’re not gonna fucking kill me or be aggressive…(Akina, pan woman, CT)*

## Discussion

Unlike most research on GSN apps, this study took a sex-positive approach and aimed to explore a broad spectrum of perceived health and wellbeing impacts of GSN apps. A range of positives and risks of using apps were reported and participants sought to balance these with a variety of strategies.

We have found that GSN app use in itself should not be perceived as a risk to health. Although continued use of the apps does not necessarily mean the perceived positive impacts outweigh the risks, only one participant reported having stopped using apps because of negative experiences. Overall, our participants reported more health and wellbeing benefits than negative outcomes. Participants were already using lots of strategies to mitigate risk and promote mental and sexual wellbeing whilst using the apps.

### Perceived benefits and positive health and wellbeing experiences

Positive health impacts of apps were reported by all participants. Social connections, sexual and romantic partners, mental wellbeing boosts and sexual pleasure were valuable positive outcomes. It is important to note, sex was only one reason people were using GSN apps. Similar to a recent survey of LGBTQIA app users in the USA (Sharabi et al., 2023), our participants of all genders in both countries reported finding community, friendship, romance, love and sex on apps. Participants also reported apps had helped them reduce loneliness and connect with their local LGBT community. Substantial evidence shows LGBTQ+ people in the UK and the USA experience worse physical and mental health than the general population (Booker et al., 2017; Zeeman et al., 2019), in part caused by discrimination and minority stress (Frost & Meyer, 2023). Social cohesion and connection to a LGBTQ+ community is linked to lower internalized homonegativity and minority stress, and improved self-esteem and long-term health outcomes (Detrie & Lease, 2007; Gibbs & Rice, 2016; Hill & Gunderson, 2015; Meyer, 2003). Recent research in Germany has suggested that GSN apps and online dating sites can aid MSM in dealing with the adverse effects of minority stress (Cargnino & Lemke, 2023); however more research is needed.

Moreover, a recent US Surgeon General report showed the effects of loneliness, caused by social isolation, has been increasing for two decades with severe impacts on health and wellbeing (Office of the Surgeon General, 2023). The report presents data (originally published by Holt-Lunstad et al., 2017) showing lack of social connection has a greater negative impact on health than smoking 15 cigarettes a day or drinking 6 alcoholic beverages per day. Our findings show GSN apps have potential to increase social capital, improve social cohesion and reduce loneliness.

To our knowledge very few primary research studies have examined the perceived health and wellbeing impacts of GSN app use using an explicitly sex-positive framework considering sexual activity, pleasure and relationships as a benefit to users. Previous public health research on how lesbian, gay, and bisexual communities use GSN apps has mainly focused on sexual disease outcomes and sexual risk-taking behavior by MSM (Choi et al., 2017; Wang et al., 2018) and the broader literature on dating apps is now beginning to examine the positive health and wellbeing impacts of apps (Castro & Barrada, 2020; Sharabi et al., 2023). GSN app use is very common, and apps are no longer a new or novel technology; they are here to stay. Public Health’s focus on risks and disease ignores the potential health and wellbeing benefits of sexual activity; sexual pleasure is a key driver of sexual activity and cannot be overlooked in health policy, healthcare delivery or health promotion (Allen & Carmody, 2012; Ford et al., 2019; Williams et al., 2015). A meta-analysis by Scott-Sheldon and Johnson (2006) showed interventions that included erotism (e.g. pleasure) reduced risky sexual behaviors and improved condom-use intentions. Judgmental or sex-negative health promotion risks alienating app users; ignoring pleasure is unrealistic and disconnected from people’s experiences, aspirations, and concerns (Ford et al., 2019). In addition, focusing exclusively on negative outcomes may further stigmatize a community that already suffers from considerable discrimination and heterosexism.

### Perceived risks and negative health and wellbeing experiences

Our research agrees with the current evidence that there are also important negative health and wellbeing impacts for GSN app users. Men in this study reported more negative experiences and health outcomes than women. Many men reported witnessing or experiencing discrimination, racism, body-shaming and homonegativity on apps – these have been reported widely in the literature (Albury et al., 2019; Filice et al., 2019; Hammack et al., 2022; Lauckner et al., 2019; Wade & Harper, 2020).

These negative experiences cannot be ignored and despite our sex-positive approach and the positive perceived impacts reported above, we must stress more work is needed to reduce these negative health and wellbeing outcomes of apps. Governance on GSN app platforms (to control racism, discrimination and offensive behavior) is insufficient and Duguay et al. (2020) argue that the companies need take more systematic approaches that consider the role of a platform’s architecture in shaping and sustaining dominant cultures on technology. Apps themselves must work harder to reduce offensive and discriminatory behavior on apps. Most apps have community guidelines, however, the difference between “sexual preferences” and “racial discrimination” is complex and understanding varies amongst app users (Callander et al., 2016). However, the discrimination experienced on apps reported by a notable proportion of our male participants could be further adding to minority stress. It is important to consider how apps could exacerbate or mitigate existing mental health inequalities. Further research is needed to investigate how the social benefits of apps can be maximized to reduce health inequalities.

### Strategies to balance risks and positive experiences

Most of the reported positive health and wellbeing impacts also had a negative flipside, and sometimes these were reported by the same person. For example, whilst the majority of participants felt apps improved their self-esteem, the majority also discussed how apps damaged their self-esteem; half of participants discussed experiencing both boosts and damage to their egos. Participants found apps allowed them to contact a wider range of potential partners; however, this came with experiences of rejection. Many people had found a community, friendship, and social support on apps, but many, mainly men had experienced discrimination, offensive behavior or homonegativity from strangers on apps.

Sex-positivity acknowledges these risks and concerns, whilst also embracing the positive outcomes of sexual pleasure, wellbeing, and freedom (Williams et al., 2015). This balance is beginning to emerge in the literature; for example, Canadian health professionals who work with young MSM report they view dating apps as a “double edged sword” (Gaudette et al., 2023) and the paradox of “disconnected connectedness” experienced by app using MSM has been discussed in the psychiatric literature (Goldenberg, 2019).

Participants reported using various strategies to protect themselves on apps. Men were more likely to talk about safer sex and women were more concerned about physical safety. In terms of mental health, men and women were similar in how they interact on apps and regularly delete apps. Three main approaches were reported: (1) participants made deliberate and conscious decisions about how they interacted with people on apps, (2) participants (mainly women) had specific strategies they employed when arranging to meet up with people in real life, and (3) deleting or uninstalling GSN apps was a common protective behavior amongst all genders.

Our findings show that app users are already using a variety of harm reduction strategies to limit their physical and mental health risks. Health promotion is most effective when it builds on what people already do, ensuring people have the capabilities, opportunities and motivations to make manageable changes (Michie et al., 2014). Indeed, the framework proposed by Williams et al. (2015) suggests sex-positivity is a strength-based approach; participants in this study reported many strengths and assets that enable them to stay happy, and healthy. We need to support people’s resources, drawing from their existing strengths to resolve problems and helping them to be happier and more fulfilled (Williams et al., 2015).

Our findings demonstrate that most GSN app users are motivated to reduce risk, and their existing strategies can provide a basis for health promotion to build on when designing sexual health programming. Resources are needed to provide guidance and support to help individuals navigate their app use while protecting their mental and physical wellbeing. App companies themselves should take some responsibility as their products have the potential to both contribute and to reduce stress and discrimination experienced by LGBTQ+ individuals. Maximizing the positive impact of these apps, to increase social cohesion while ameliorating mental health inequalities, requires further research.

### Cross-cultural comparison

Understanding variations between countries and cultures can help us tailor health promotion interventions to be more culturally appropriate and effective (Al-Bannay et al., 2014). We found there was very little difference between the participants from the UK and USA in perceived health impacts or strategies to stay healthy. The global nature of GSN app technology and the ubiquity of Grindr (and other apps) is likely influencing social norms in diverse countries and communities (Cserni, 2020). Although this is a small study, our findings suggest many of the health and wellbeing experiences reported in the research with MSM in North America, Australia, and China are likely to be experienced by MSM in the UK. Additional cross-cultural research, with participants from a wider range of countries (including low- and middle-income countries) and across LGBTQ+ population groups, is needed to explore this further.

The political and cultural context in the USA has changed dramatically recently. This data was collected during President Donald J. Trump’s first term (2017–2021). However, early in his second term (2025 to date), President Trump has implemented a number of executive orders that have impacted LGBTQ+ populations in the USA, particularly those who are trans and non-binary. Public health organizations have highlighted the threat to public health and, in particular, to the health of gender and sexual minorities in the USA (American Public Health Association, 2025). The differences between the participants in the UK and USA would likely be starker if this data were collected in 2025. However, the rise of the Far Right has also been highlighted as a risk to the health of LGBTQ+ people globally (March et al., 2025).

Although this was a small-scale exploratory study, the findings do add to the evidence on app use by sexual minorities in different countries, and provides preliminary evidence it may be similar, at least in our two areas. The only other qualitative research we found on GSN app use by sexual minorities from multiple countries combined two separate interview studies conducted in Australia, Canada, and the United Kingdom (Ferris & Duguay, 2020). In this, the authors investigate self-presentations of sexual identity on Tinder and do not explore any differences *between* the countries. Our study used the same interview guide and photo-elicitation prompts to explore the behavior in two countries, and explore any differences in our sample.

### GSN app use by women

There is a dearth of research on GSN app use by sexual minority women (Castro & Barrada, 2020) and to our knowledge this is the first qualitative research study about GSN apps that has included both sexual minority men and women. The perceived health and wellbeing impacts of GSN app use were similar for men and women in our study; however, the negative health impacts were more likely to be experienced by men.

In this study women generally reported more positive experiences, and the risks tended to focus on self-esteem and rejection. Despite women participants in this study reporting fewer risks or negative outcomes, they utilized more measures to maintain personal safety, especially plurisexual women who were meeting men. Previous research with heterosexual women has shown experience of sexual harassment and dating violence are common on apps (Bates et al., 2023; Castro & Barrada, 2020). A recent scoping review found women and sexual minorities are at higher risk of experiencing harassment on GSN apps (Gewirtz-Meydan et al., 2024). Experiences of violence and harassment were rarely mentioned by women in our study, and when they were it related to men. Most plurisexual women in this study did not use apps to meet men for this reason.

The women who participated in this study reported fewer negative experiences of GSN apps when they used them to meet women, compared to meeting men. For the women in this study who had only had same-gender partners, apps were perceived as having a mainly positive impact on health and wellbeing. Experiences of plurisexual women need further investigation, especially given bisexual and other plurisexual persons experience worst health inequaltiies, compared to lesbian, gay, or heterosexual people (Booker et al., 2017; Colledge et al., 2015; Dilley et al., 2010).

### Limitations

The main potential limitation in this research comes from the recruitment methods and sampling. The sample was highly educated, and many worked at or studied at universities. Their experiences are likely to differ from those of more deprived and less educated groups. Although the participants were predominantly white, in both areas they were relatively representative of the local population. The sample was not large with recruitment focused only on one area in each country. The findings are unlikely to be generalizable to the whole population of USA and UK, but might be transferable to similar areas.

As with all qualitative methodologies, the researcher themselves may introduce bias. The researcher’s beliefs, position, or opinions may impact the course of the interviews or the interpretation of participants responses. The semi-structured interview guide and photo-elicitation method added replicability, and codes/themes were discussed with coauthors at three stages of the analysis.

The data were collected before the global COVID-19 pandemic and behavior likely changed during lockdowns and then again as restrictions were removed. Although Donald Trump was the US President when these data were collected, the cultural situation for LGBTQ+ people in the USA has changed dramatically since he came back into office in 2025.

Approximately half of the interviews were conducted in coffee shops; all participants were offered the option to attend a university building, a café or choose their own venue. Those who chose a café explained that it was more convenient and they felt more relaxed in the café. Despite this, a public interview venue may have influenced how comfortable the participants felt and may have affected how open and candid they were.

It must be acknowledged this research focused on sexual orientation (e.g. those who identified as lesbian, gay, bisexual, pansexual, etc., and those seeking same-gender partners) and we were not able to include a wide variety of gender diverse participants. One trans lesbian participated; however, non-binary participants were not included. Further research is needed to understand the complex experiences of a more gender diverse sample.

## Conclusion

Repeated research has shown sexual minorities experience worse mental and physical health; however, this novel study has shown that GSN apps have the potential to provide positive sexual experiences, improve mental health and increase social cohesion for both men and women. Taking a sex-positive approach encourages us to examine both positive and negative perceived health outcomes, and to consider the strengths and assets people have that promote wellbeing. This study has also highlighted concerns about the potential negative impact of app use on health and wellbeing. The majority of participants were taking measures to protect their health and for most the positives outweighed the negatives. For women in this study who only dated/had sex with women, apps were thought to be having a mainly positive impact on health and wellbeing.

Finally, the study found similarities in risks, perceived impacts and strategies of use between the USA and UK, highlighting the global nature of GSN app technology. To our knowledge this is the first qualitative study investigating cross-cultural differences in GSN app use by this population. These findings provide preliminary evidence that GSN app social norms are likely to be similar across diverse countries and communities.

We would like to see a shift in focus from public health research which solely examines risks, to research which acknowledges the potential positive health and wellbeing impacts of sexual pleasure, friendships, love and community connections found through GSN apps. We must also focus on the strengths, assets and resources individuals already use to promote health. Sex-positive health promotion can help reduce stigma and discrimination faced by LGBTQ+ communities and we need a more inclusive approach to understanding app use.

## Supplementary Material

Supplemental Material

## Data Availability

The participants of this study did not give written consent for their data to be shared publicly, so due to the sensitive nature of the research supporting data is not available.

## Note

1 The number of avenues, routes or technologies used to find sexual partners (e.g. GSN apps, saunas/bathhouses, bars, cruising areas, etc.) is related to risk. Those using multiple ways to find partners were more likely to test positive for an STI and report risky sexual behaviour (including condomless anal intercourse, group sex and sex tourism).
