# Supplementary material for: Moving Beyond “Risky Behavior”: A Qualitative Interview Study Exploring Geosocial Networking App Use by Sexual Minority Men and Women in the UK and USA
Source: Int J Sex Health. 2025 Jul 28;37(3):503–24. doi: 10.1080/19317611.2025.2536248 (PMC12366820; doi:10.1080/19317611.2025.2536248)
Supplement: Supplemental Material [file WIJS_A_2536248_SM9139.docx]

**Supplementary file 2: details of the prompts used during the photo-elicitation phase of the interviews**

A UK and US version was produced for each fake profile – photos and text remained very similar however, place/university names and units of distance were changed to be relevant to that locality. The profiles were printed out in colour and laminated.

**Women’s fake profiles:** The women’s profiles were designed to cover a variety of typical profiles seen on Tinder. At the time, Tinder profiles had to include a photo, first name and age; the information about education and job were optional.


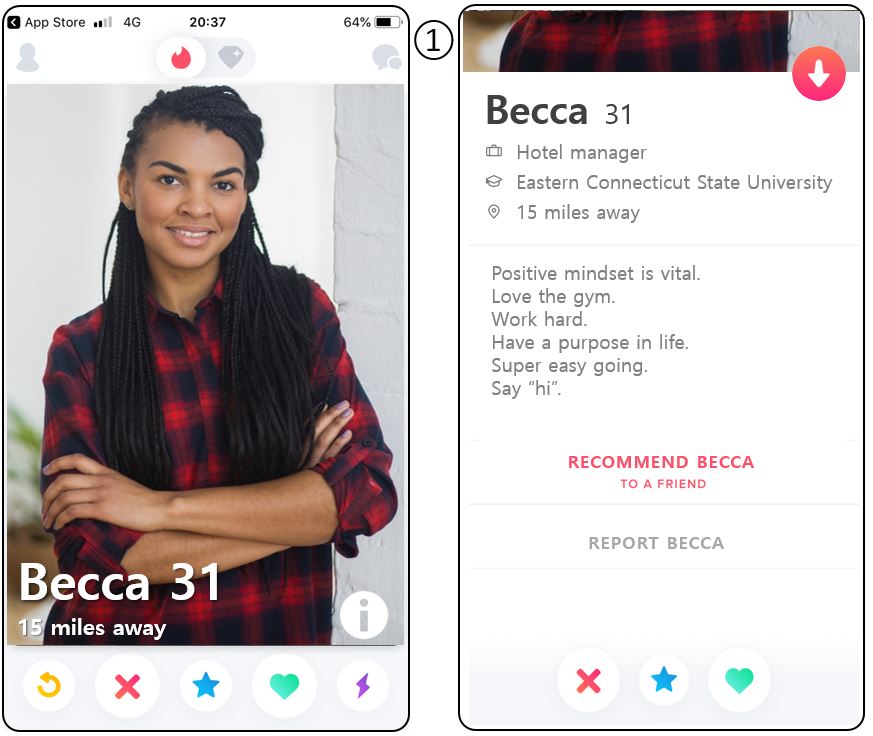


[Image: Freepik.com]


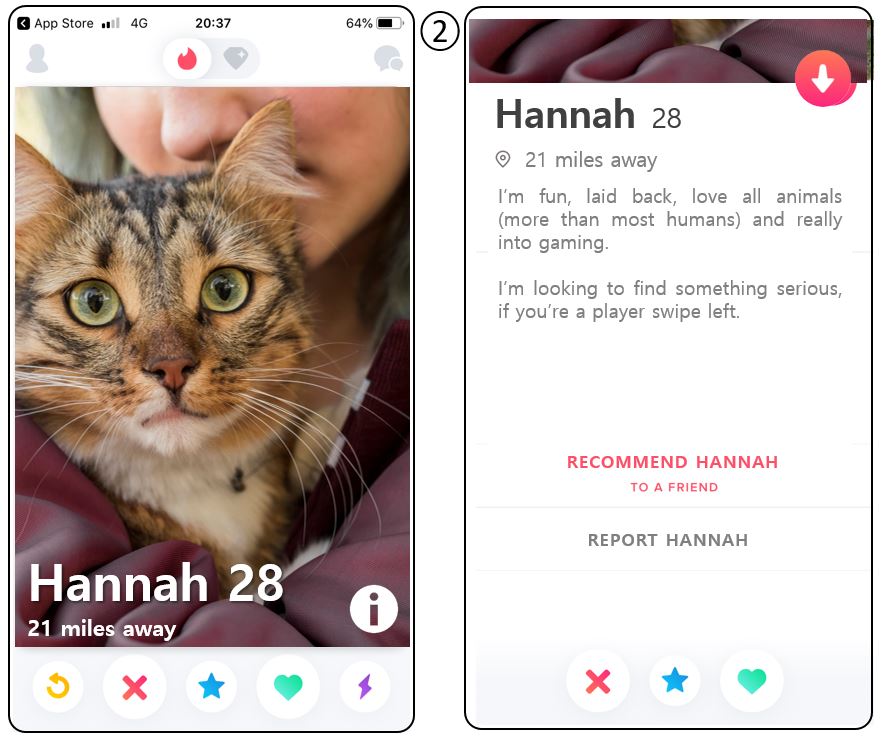
 [Image: Freepik.com]


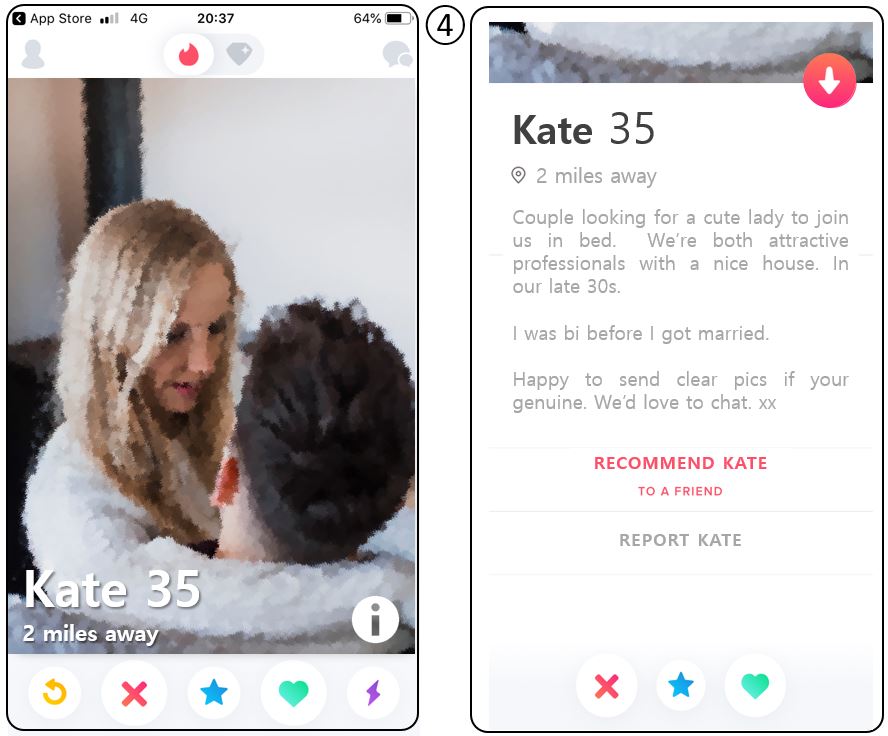


[Image: Freepik.com]


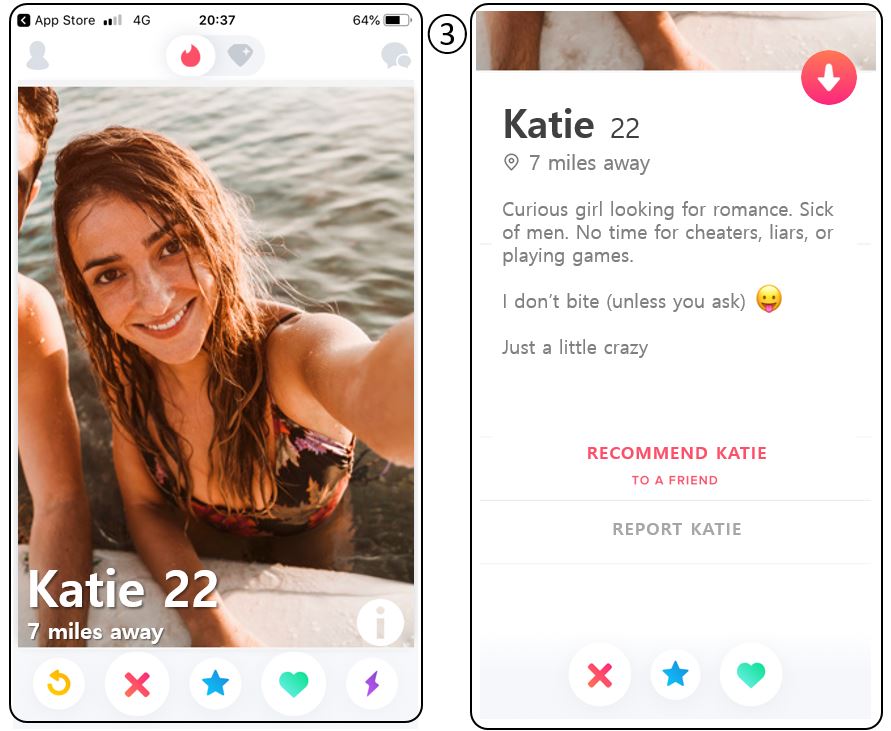


[Image: Freepik.com]

**Men’s fake profiles:** The men’s profiles show a range of typical profiles on Grindr. At the time, Grindr users were not required to have a photo or age, and all fields are optional. The username could be their first name, a word or phrase (including emojis). The profiles of men were more sexually explicit and reflect the more sexual nature of Grindr. Grindr profiles include acronyms, which were also discussed.


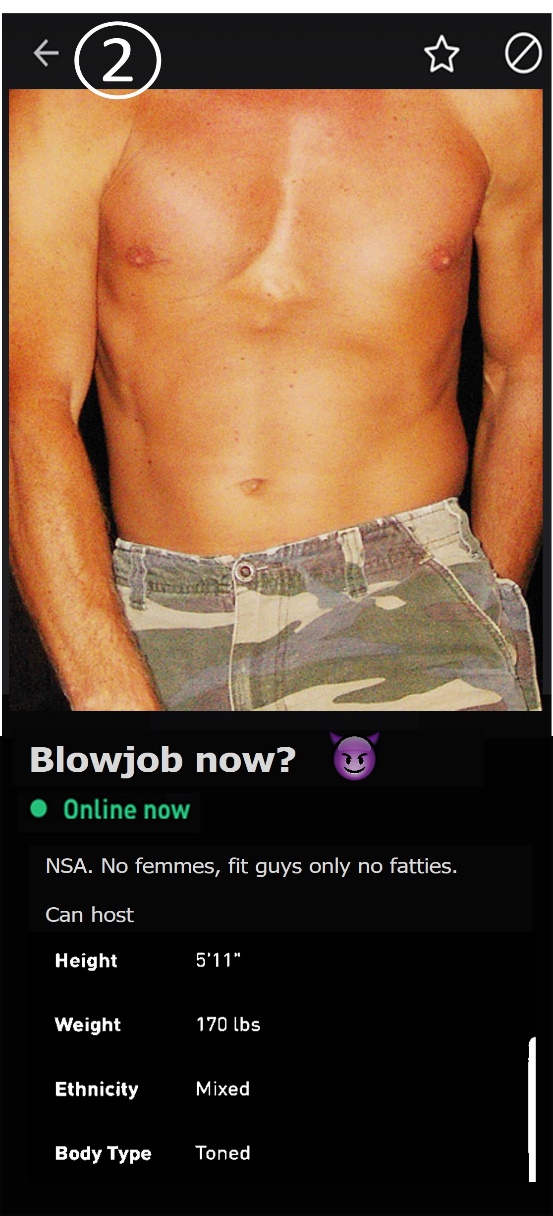

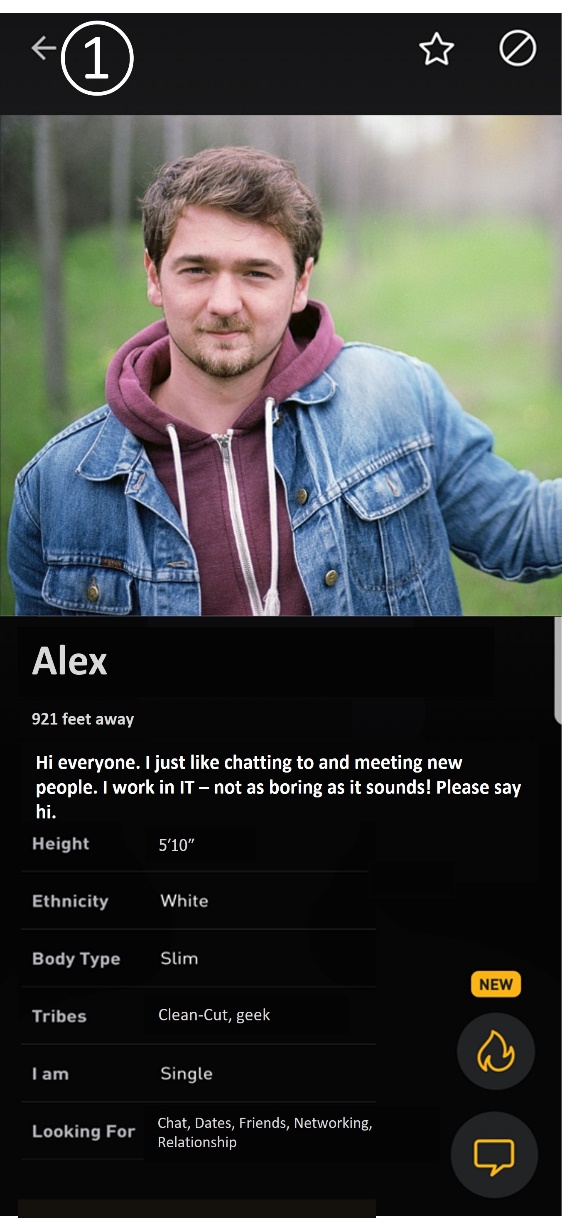


[Image: pxhere.com] [Image: Morne, Public domain, via Wikimedia Commons]


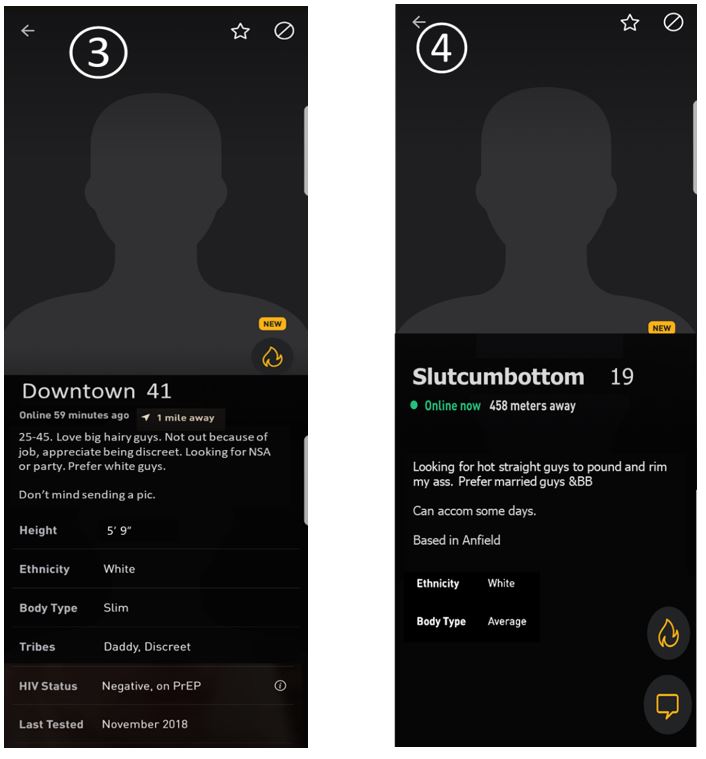


[Images generated by author]
